# Principles of Human Physiology, with Their Chief Applications to Pathology, Hygiene, and Forensic Medicine

**Published:** 1847-12

**Authors:** 


*Principles of Human Physiology, with their chief applications to Pathology, Hygiene, and Forensic Medicine.* By Wm. B. Carpenter, M. D., F. R. S. Fullenian Professor of Physiology in the Royal Institution of Great Britain, Corresponding Member of the American Philosophical Society, and of the National Institute of the United States, &c.. &c. Third American, from the last London edition. With notes and additions, by Meredith Clymer, M. D., Consulting Physician to the Philadelphia Hospital, late Professor of the Principles and Practice of Medicine and Clinical Medicine in the Franklin Medical College, Philadelphia, etc., etc. With three hundred and seventeen wood cuts and other illustrations. Philadelphia, Lea and Blanchard: 1847; 8vo. p.p. 752.

That our readers may not confound the different publications emanating from the pen of Dr. Carpenter, we should state, that this is not the work noticed by us several months ago. The latter was entitled *Elements of Physiology*, and differs from the present in being of a more elementary character. His work on the *Principles of Physiology*, which is more comprehensive in its scope, first appeared four years ago, and having passed, in the mean time, in Great Britain through three editions, the present, as the reader will perceive by the title page, is the third American, reprinted from the latest London edition.

Physiology is eminently a progressive science. Under the combined influences of microscopical researches, and the application of organic chemistry, its character has materially changed within a few years. Animating as is the contemplation of scientific advancement, it is sometimes disheartening to reflect, that when we have mastered all that appertains to a particular stage of progress, our future labors are in no wise lessened. That imaginary elysium of repose which stimulates the industry of those who strive for wealth, can have no place, even in the fancy of the devotee of science. The fruits of the exertions of the latter consist in acquiring greater inducements and facilities for continued application. He is satisfied to have it so: it is only in his occasional moments of fatigue and discouragement, that he feels dissatisfied with the toil which his pursuits impose upon him.

Assuredly, no province of scientific truth can boast of more brilliant epochs than have characterized the history of Physiology for the last quarter of a century! The Practitioner who entered upon the study of medicine less than twenty years ago, must retain a vivid recollection of the *then* philosophical works of Bichat, Richerand and Hunter. But contrast the present condition of the science with what it was in the possession of the vast imprrovements which emanated from these and other contemporaneous physiologists! Consider the discoveries of Sir Charles Bell, and the later advances made in the knowledge of the nervous system by Marshall, Hall, and others; the development of the cell philosophy; the splendid results of analytical chemistry, and the boundless anticipations which may be rationally entertained of its future disclosures —these, and other characteristics of progress only carry us back but a few years, within the memory of those of us who are among the junior members of the profession; and we have every reason to believe, that the events of the next few years will not be of a less striking and important character.

Commensurate with the constant acquisition of physiological knowledge, have been useful additions to Pathology and Therapeutics. This must ever be the case, so intimate are the relations existing between the study of life in its healthy, and in its morbid manifestations. Hence it is, that the practitioner who would keep pace with the progress of medicine, must not fail to make himself familiar with the important facts which are constantly being educed by the labors of the physiologist. The practical necessity of the study of physiology derived from its close relationship to the other departments of medical science, is so clear, and so generally appreciated, that its discussion would be gratuitous.

These considerations are not inappropriately introduced in a notice of the work whose title page is prefixed to this article; for in this work the student and practitioner will find a lucid, philosophical, and sufficiently comprehensive exposition of physiological science as it exists at the present moment. More than this we need not say, as we have heretofore taken occasion to express at some length our views of Dr. Carpenter as a physiological writer [Review of *Elements of Physiology* in vol. 2d of this Journal].

With reference to the merits of this edition of the work, it is but fair to present the following quotations from the Preface:—

"The present edition has not only undergone a very careful revision; but has in many parts received large additions, and has been in many others entirely remodelled." * * "The quantity of matter has been augmented by an amount equivalent to the addition of fully a hundred pages. A considerable number of new wood engravings of first rate execution, have, also, been introduced. Among the principal additions will be found a chapter on the varieties of the human race; intended to convey to those, who have not time or opportunity to peruse Dr. Prichard's elaborate treatises, a general view of his arguments and conclusions. The account of the Primary Tissues, which was formerly included in the chapter on Nutrition, has been extended in a degree commensurate with the Author's estimate of its importance, and has been made to form a distinct chapter nearer the commencement of the work." * * "Many, however, of the additions, and alterations scattered through the work, are the result of the Author's own investigations."

By the Preface of the American Edition we learn, that many of his notes to previous editions have been incorporated, by the Author, in the text of the present edition—which is not stated by Dr. Carpenter, as, it seems to us, should have been done, if he considered them of value, and was indebted for the facts which they contain to Dr. Clymer. The American edition, also, contains one hundred and fifteen more wood cuts than the third English edition.

That these additions possess value we do not deny, yet we must confess that we should have been better pleased if the duties of the American Editor had been confined to supplying omissions of the contributions of our own countrymen, and in this respect, we are sorry to say, the work is defective. We looked in vain through the section treating of animal heat, and the chapter on muscular contractility, for reference to the striking observations of Dr. Dowler, of New Orleans. These should not have escaped the notice of the Author, but it is quite inexcusable that they should have been silently passed over by the American Editor.

In justice to the candor of Dr. Carpenter it deserves mention, that he does not hesitate to accord to Dr. Moultrie, of Charleston, S. C., the credit of the hypothesis that the final purpose of lymphatic absorption is for nutrition, not excretion; a hypothesis which Dr. C. had supposed was original with himself, before meeting with the writings of Dr. M. on that subject. We are also gratified to perceive that he adopts the very ingenious and philosophical explanation of the forces presiding over capillary circulation offered by Prof. Draper of the University of the city of New-York.

We conclude by commending the work most heartily to our readers. In mechanical execution it is in the usual style of Lea and Blanchard.

